# The multiple mediating effects of self-efficacy and resilience on the relationship between social support and procrastination among vocational college students: a cross-sectional study

**DOI:** 10.1186/s12889-024-19487-6

**Published:** 2024-07-23

**Authors:** Yanting Zhang, Hongyu Guo, Mei Ren, Haili Ma, Yingying Chen, Cancan Chen

**Affiliations:** 1 School of Nursing, Zhengzhou Railway Vocational & Technical College, Zhengzhou, Henan China; 2 Department of Nursing, Henan Provincial Key Medicine Laboratory of Nursing, Henan Provincial People’s Hospital, Zhengzhou University People’s Hospital, 7# Weiwu Road, Jinshui District, Zhengzhou City, Henan Province 450000 China

**Keywords:** Procrastination, Social support, Self-efficacy, Resilience, Vocational college students

## Abstract

**Background:**

Previous research has revealed a negative association between social support and procrastination. However, few studies have investigated the mechanism underlying this relationship among vocational college students.

**Objective:**

Based on the social cognitive theory, this study was intended to investigate the multiple mediating effects of self-efficacy and resilience on the relationship between social support and procrastination among vocational college students.

**Methods:**

This study employed a cross-sectional design involving a sample of 1,379 students from a vocational college in China. Data were collected using the General Procrastination Scale, the Multidimensional Scale of Perceived Social Support, the General Self-Efficacy Scale, and the Resilience Scale-14. The PROCESS macro for SPSS was used to examine the multiple mediation model.

**Results:**

Our findings indicate significant negative correlations between social support, self-efficacy, resilience, and procrastination. The multiple mediation analysis showed that social support did not have a significant direct impact on procrastination. Instead, the relationship between social support and procrastination was fully mediated by self-efficacy (indirect effect: -0.017; 95% CI: -0.032, -0.004) and resilience (indirect effect: -0.047; 95% CI: -0.072, -0.025), and sequentially mediated by both factors (indirect effect: -0.013; 95% CI: -0.020, -0.007).

**Conclusions:**

The results emphasise the importance of enhancing self-efficacy and resilience in initiatives aimed at preventing and intervening in case of procrastination among vocational college students. Additionally, strengthening social support may also be crucial to preventing or reducing procrastination among this population.

## Introduction

Procrastination has been defined as the voluntary delay of an intended course of action although the delayer expects to be worse off as a result [1]. Procrastination is highly prevalent among college students, with an estimated rate of 80–95% [2]. Most students who engage in such behaviour view it as inappropriate, problematic, and needing to be changed [3]. Furthermore, procrastination often results in lower academic performance, increased distress, and decreased physical and mental health [4–6]. Various studies have investigated the factors that contribute to procrastination, including individual characteristics such as personality traits [7], emotions [1, 8], and cognitive beliefs [9, 10], and environmental factors such as social support [11, 12], parenting styles [13, 14], and environmental unpredictability [15]. However, few studies have examined both environmental and personal factors, or the underlying mechanisms informed by theoretical frameworks.

**Fig. 1:**
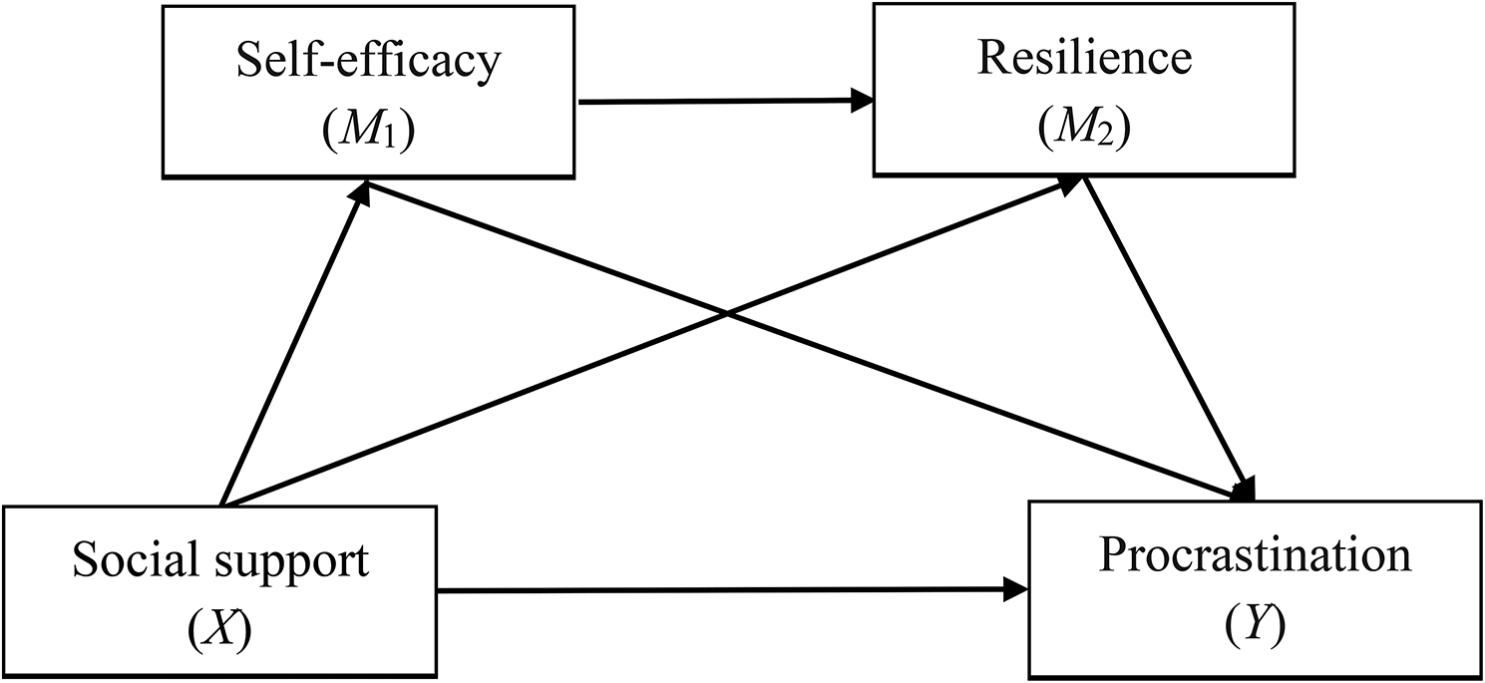
The hypothetical mediation model of social support on procrastination

Procrastination has been described as a form of self-regulatory failure [1, 16], characterised by an individual’s failure to take action (underregulation) or by the taking of ineffective action (misregulation) when attempting to initiate, alter, or inhibit a behaviour [17]. Social Cognitive Theory (SCT) is distinctive in viewing self-regulation as an interaction of environmental, personal, and behavioural triadic processes [18, 19]. SCT proposes that both environmental and personal factors influence behaviour [19]. Within this framework, environmental factors (e.g., social support) [20, 21] and personal factors (e.g., self-efficacy and resilience) [22–24] interact to motivate and regulate human behaviour [25, 26]. Furthermore, personal factors can serve as mediators between environmental factors and behaviour [27, 28]. This model may aid researchers in identifying modifiable factors required to prevent or diminish undesired behaviours (e.g., procrastination), thereby supporting the development of behaviour change interventions. Building upon the SCT and prior research, we operationalised social support as an environmental factor, and self-efficacy and resilience as personal factors, with procrastination representing the behaviour. We sought to investigate the potentially mediating roles of self-efficacy and resilience in the association between social support and procrastination among vocational college students. The proposed mediation model is depicted in Fig. 1.

### Social support and procrastination

Social support has been conceptualised as the material, emotional, informational, and instrumental assistance and resources individuals receive from their social networks, encompassing family, friends, and the community [29]. Scholars have suggested that social support – an essential environmental factor of the SCT [30] – is an important factor in influencing procrastination [11, 31]. Ferrari et al. [31] investigated the social support networks of procrastinators and indicated that habitual procrastination could be linked to poor family relations and disrupted or unsatisfactory social relationships. In other words, dependable social support networks can provide individuals with valuable perspectives, advice, and problem-solving strategies, and help motivate them to take action to solve problems and pursue their goals, thereby helping them prevent or overcome procrastination [12, 32]. Madjid et al. [12] found that social support from family, friends, and school could decrease the degree of academic procrastination among university students. Yang et al. [11] demonstrated that social support was a negative predictor of procrastination among college students. However, research on the impact of social support on procrastination among vocational students is limited, and few studies have examined the mechanism whereby social support – an important external environmental factor – translates into less procrastination behaviour. On the basis of the SCT [19], we sought to explore whether personal factors (self-efficacy and resilience) play mediating roles in the relationship between social support and procrastination.

### The potential mediating effect of self-efficacy

Self-efficacy is defined as ‘the belief in one’s capabilities to organize and execute the courses of action required to manage prospective situations’, which influences the way an individual thinks, feels, and behaves [33]. According to the SCT, self-efficacy is a key component of personal factors and plays a central role in the exercise of personal agency and behaviour change [34, 35]. Self-efficacy determines the level of effort individuals will exert in a task, their persistence in overcoming obstacles, and whether their cognitive patterns are self-limiting or self-enhancing in dealing with environmental challenges [25]. Individuals with low self-efficacy perceive themselves as incapable of managing situations, often avoid challenging circumstances [35], and are more prone to procrastination [36]. Several studies have demonstrated the negative impact of self-efficacy on procrastination [1, 9, 37]. A meta-analysis of 39 studies (*N* = 6,994) revealed that self-efficacy was significantly correlated with procrastination, with an average effect size of -0.38 [1]. Self-efficacy has also been identified as a negative predictor of procrastination [9, 37]. In addition, self-efficacy has been found to be positively predicted by social support [38, 39]. High levels of social support can provide individuals with resources, guidance, and encouragement, which help enhance individuals’ beliefs in their capacity to deal with challenges, thus reinforcing their self-efficacy [39]. Hence, it is plausible to suggest that students’ perceived social support is linked to their self-efficacy, which in turn, could influence their propensity to engage in procrastination. Therefore, we assume that self-efficacy can serve as a potential mediator between social support and procrastination.

### The potential mediating effect of resilience

Resilience refers to ‘manifested competence in the context of significant challenges to adaptation or development’ [40]. Resilience is a crucial factor linked to procrastination within the context of social interactions [41]. Social support serves as a positive predictor of resilience and has a beneficial impact on it [42, 43]. Social factors such as strong familial bonds and supportive relationships play a significant role in the development and cultivation of resilience [44]. Moreover, resilience has been found to be beneficial in the prevention and reduction of procrastination [41, 45]. Ko and Chang [41] have reported that students with stronger degrees of resilience had a lower tendency to procrastinate. Shin and Kelly [45] found that college students who reported greater resilience were less likely to procrastinate in their career decision making. Thus, resilience may be a mediator between social support and procrastination.

### The potential serial mediation role of self-efficacy and resilience

According to Bandura [25], individuals’ self-efficacy can influence their resilience and bouncebackability following setbacks or failures. Individuals with greater self-efficacy tend to exhibit better resilience when faced with adversity [46]. Schwarzer and Warner [47] demonstrated that self-efficacy bolstered resilience by activating emotional, motivational, and behavioural processes in challenging situations. Sabouripour et al. [48] reported that self-efficacy positively predicted resilience among university students. Given the role that social support can play in strengthening self-efficacy in close association with resilience, which negatively predicts procrastination, we believe that social support has an indirect impact on procrastination by influencing self-efficacy and, subsequently, resilience. Therefore, we deduce that self-efficacy and resilience serve as sequential mediators between social support and procrastination.

### The present research

This study was intended to identify the impact of social support on procrastination and explore the intermediary mechanism of self-efficacy and resilience between them among vocational college students. The proposed hypotheses are as follows:

#### H1

*Self-efficacy serves as a mediator between social support and procrastination among vocational college students.*

#### H2

*Resilience serves as a mediator between social support and procrastination among vocational college students.*

#### H3

*Self-efficacy and resilience act as sequential mediators between social support and procrastination among vocational college students.*

## Methods

### Design and sampling

We conducted a cross-sectional study using convenience sampling of students from a vocational college in Henan, China. A total of 1,379 vocational college students voluntarily participated in and completed our survey. The average age of the participants was 19.93 years (SD = 1.32), 740 (53.7%) were males, and 639 (46.3%) were females. In addition, 523 (37.9%) were first-year students, 618 (44.8%) were second-year students, and 238 (17.3%) were third-year students.

### Procedure

Upon receiving approval from the ethics review committee of our college, we used the ‘SOJUMP’ online survey platform (a professional data platform in China) to collect data in early March 2021. The survey link was distributed to nursing student counsellors, who then shared it with their students, along with the details about the study’s objectives, content, anonymity, and confidentiality. Students voluntarily participated in the survey by accessing the link and completing the questionnaire online. To enhance response validity and prevent omissions and duplicates, students were instructed to follow a standardised completion protocol at the survey’s outset, and submission was only permitted after each item was addressed, with no opportunity to revise responses post-submission. The act of submitting a completed questionnaire was construed as implicit consent.

### Measures

#### General procrastination scale (GPS-9)

Procrastination was evaluated using the Chinese version of the GPS-9 [49], which is the short form of Lay’s General Procrastination Scale developed in 1986 [50] and adapted by Sirois et al. in 2019 [51]. The GPS-9 contains nine items that assess trait procrastination in various daily tasks (e.g., ‘I often find myself performing tasks that I had intended to do days before’). All items were answered on a 5-point Likert scale (1 = strongly disagree, 5 = strongly agree), and the total score ranged from 9 to 45. Of the nine items, three were subject to reverse scoring; after the scores were reversed, higher total scores reflected a greater tendency to procrastinate. The GPS-9 has shown adequate validity and reliability [49, 51, 52], and its internal consistency was α = 0.730 in this sample.

#### Multidimensional scale of perceived social support (MSPSS)

Social support was assessed using the Chinese version of the MSPSS [53], which was developed by Zimet in 1988 [54]. The MSPSS consists of 12 items that assess three dimensions: (1) perceived family support, such as ‘I can talk about my problems with my family’; (2) perceived support from friends, such as ‘I have friends with whom I can share my joys and sorrows’; and (3) perceived support from significant others, such as ‘I have a special person who is a real source of comfort to me’. Participants responded to all items using a 7-point Likert scale (1 = very strongly disagree, 7 = very strongly agree). The total score ranged from 12 to 84, and higher scores reflected greater levels of perceived social support. The MSPSS has shown good validity and reliability [53, 55, 56], and its internal consistency was α = 0.971 in this sample.

#### General self-efficacy scale (GSES)

Self-efficacy was evaluated by the Chinese version of the GSES [57], which was compiled by Schwarzer in 1993 [58]. The GSES contains 10 items assessing individuals’ beliefs in their capability to effectively cope with diverse challenging environmental demands (e.g., ‘I can usually handle whatever comes my way’). For each item, participants chose an answer on a 4-point Likert scale (1 = not at all true, 4 = exactly true). The total score ranges from 10 to 40, with higher scores indicating better self-efficacy. The GSES has demonstrated satisfactory validity and reliability [57, 59, 60], and its internal consistency was α = 0.949 in this sample.

#### Resilience scale-14 (RS-14)

Resilience was measured using the Chinese version of the RS-14 [61], which is the short form of Wagnild and Young’s Resilience Scale developed in 1993 [62] and adapted by Wagnild in 2009 [63]. The RS-14 contains 14 items assessing two dimensions: (1) personal competence, such as ‘I am determined’; and (2) acceptance of self and life, such as ‘I am friends with myself’. Participants responded to all items using a 7-point Likert scale (1 = strongly disagree, 7 = strongly agree). The total score ranged from 14 to 98, and higher scores indicated greater resilience. The RS-14 has demonstrated favourable validity and reliability [61, 63, 64], and its internal consistency was 0.972 in this sample.

### Data analysis

We conducted data analyses using SPSS version 26.0, with the statistical significance level set at 0.05. We computed descriptive statistics using means (standard deviations) and frequencies (percentages) and examined bivariate correlations using Pearson’s correlation analysis. We used hierarchical linear regressions to examine the association between social support, self-efficacy, resilience, and procrastination while controlling for the potential effects of age, gender, and grade. Model 1 included age, gender, and grade; Model 2 included age, gender, grade, and social support; and Model 3 included age, gender, grade, social support, self-efficacy, and resilience.

We examined the hypothesised mediation model using the Hayes PROCESS macro for SPSS, specifically Model 6 [65] – a serial multiple mediator model that assesses the effects of *X* on *Y* through four pathways: three specific indirect effects and one direct effect. One indirect pathway of *X* on *Y* went through Mediator 1 (*M*_1_) only, and the effect was *a*_1_*b*_1_. The second indirect path went through Mediator 2 (*M*_2_) only, and the effect was *a*_2_*b*_2_. The third indirect path went through both *M*_*1*_ and *M*_2_ in serial with *M*_1_ affecting *M*_2_, and the effect was *a*_1_*d*_21_*b*_2_. The total indirect effect (*ab*) was *a*_1_*b*_1_ + *a*_2_*b*_2_ + *a*_1_*d*_21_*b*_2_. The direct effect of *X* on *Y* without running through *M* was *c*’. By combining the total indirect effect (*ab*) and the direct effect (*c*’), we derived the total effect (*c*). In this study, social support was set as *X*, self-efficacy as *M*_1_, resilience as *M*_2_, and procrastination as *Y*. We evaluated the direct and indirect effects using the 95% bootstrap confidence interval (CI) based on 5,000 bootstrap samples. The effects were regarded as significant if the 95% CI did not contain zero.

## Results

### Descriptive statistics and correlations

As shown in Table 1, the mean scores for social support, self-efficacy, resilience, and procrastination were 59.22 ± 14.08, 24.76 ± 6.74, 67.54 ± 15.43, and 22.33 ± 5.61, respectively. Higher levels of social support (*r* = -0.272, *p* < 0.01), self-efficacy (*r* = -0.262, *p* < 0.01) and resilience (*r* = -0.324, *p* < 0.01) were correlated with lower procrastination tendencies. Moreover, the four variables were significantly correlated with one another.

**Table 1:** Descriptive statistics and bivariate correlations among variables (*N* = 1,379)

Variables	Mean ± SD	1	2	3	4	5	6
1. Age	19.93 ± 1.32	1					
2. Gender	0.46 ± 0.50	-0.144^**^	1				
3. Grade	1.79 ± 0.71	0.576^**^	-0.106^**^	1			
4. Social support	59.22 ± 14.08	0.033	-0.003	0.071^**^	1		
5. Self-efficacy	24.76 ± 6.74	0.077^**^	-0.129^**^	0.087^**^	0.491^**^	1	
6. Resilience	67.54 ± 15.43	0.077^**^	-0.086^**^	0.107^**^	0.734^**^	0.605^**^	1
7. Procrastination	22.33 ± 5.61	-0.097^**^	0.082^**^	-0.110^**^	-0.272^**^	-0.262^**^	-0.324^**^

Note: M = mean, SD = standardized deviation. ^**^: *p* < 0.01

### Hierarchical linear regression analysis of procrastination

We conducted a hierarchical linear regression analysis to identify the variables associated with procrastination (Table 2). After we controlled for age, gender, and grade, Model 2 revealed that social support was negatively associated with procrastination (β = -0.266, *p* < 0.01). When self-efficacy (β = -0.089, *p* < 0.01) and resilience (β = -0.206, *p* < 0.01) were included in Model 3, the relationship between social support and procrastination was not statistically significant (β = -0.072, *p* > 0.05), which indicated that self-efficacy and resilience served as mediators in the relationship between social support and procrastination.

**Table 2:** Hierarchical regressions for procrastination (*N* = 1,379)

	Model 1			Model 2			Model 3		
	β	t	*p*	β	t	*p*	β	t	*p*
Age	-0.040	-1.228	0.220	-0.044	-1.374	0.170	-0.036	-1.151	0.250
Gender	0.061	2.163	0.031	0.061	2.269	0.023	0.033	1.231	0.219
Grade, freshmen (reference: junior)	0.119	2.581	0.010	0.091	2.049	0.041	0.082	1.870	0.062
Grade, sophomore (reference: junior)	0.090	2.158	0.031	0.078	1.924	0.055	0.079	1.994	0.046
Social support				-0.266	-10.318	0.000	-0.072	-1.925	0.054
Self-efficacy							-0.089	-2.779	0.006
Resilience							-0.206	-5.005	0.000
*R* ^2^	0.019			0.090			0.123		
Adjusted *R*^2^	0.016			0.086			0.119		

Note: β = standardized beta

### Multiple mediating effects of social support and resilience

**Fig. 2:**
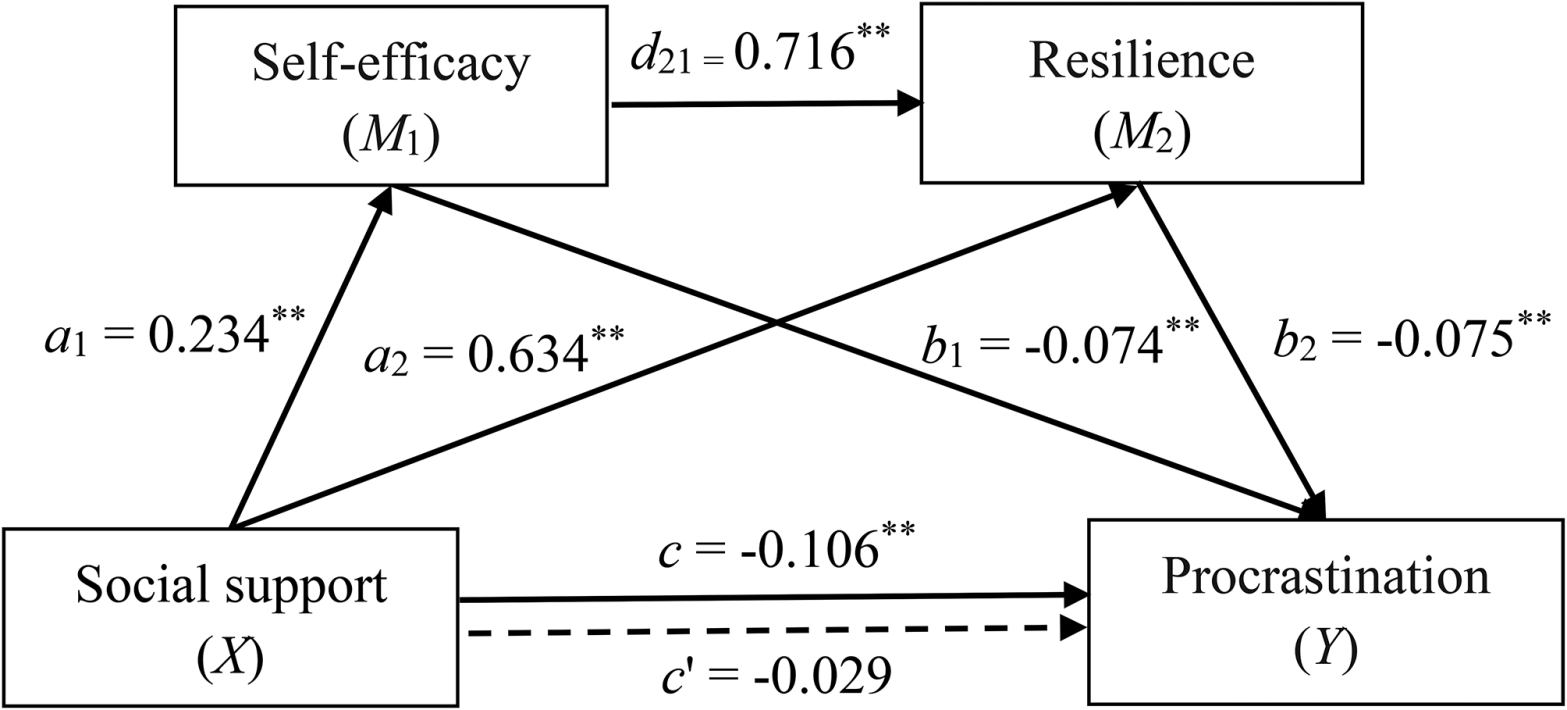
The serial multiple mediating model of social support on procrastination.* Note*: ^**^: *p* < 0.01

Hayes’ serial mediation model (Model 6) was adopted to investigate the multiple mediating roles of self-efficacy and resilience in the relationship between social support and procrastination among vocational college students. Figure 2 presents the multiple mediation model between social support and procrastination. As expected, social support significantly positively predicted self-efficacy and resilience (*a*_1_ = 0.234, *p* < 0.01; *a*_2_ = 0.634, *p* < 0.01), self-efficacy significantly positively predicted resilience (*d*_21_ = 0.716, *p* < 0.01), and self-efficacy and resilience significantly negatively predicted procrastination (*b*_1_ = -0.074, *p* < 0.01; *b*_2_ = -0.075, *p* < 0.01). Social support did not significantly predict procrastination (*c*’ = -0.029, *p* > 0.05).

As Table 3 shows, the total effect of social support on procrastination was significant (*c* = -0.106; 95% CI : -0.126, -0.086). The total indirect effect was also significant (*ab* = -0.077; 95% CI: -0.104, -0.051), with all three indirect effects showing significance. Social support indirectly affected procrastination through self-efficacy (*a*_1_*b*_1_ = -0.017; 95% CI: -0.032, -0.004) and resilience (*a*_2_*b*_2_ = -0.047; 95% CI: -0.072, -0.025), accounting for 22% and 61% of the total indirect effect, respectively. Furthermore, social support indirectly affected procrastination through self-efficacy and resilience in serial (*a*_1_*d*_21_*b*_2_ = -0.013; 95% CI: -0.020, -0.007), accounting for 17% of the total indirect effect. Moreover, social support did not directly affect procrastination in a statistically significant way (*c*’ = -0.029; 95% CI: -0.058, 0.001).

**Table 3:** Testing the pathways from social support to procrastination (*N* = 1,379)

Model pathway	Effect	BootSE	BootLLCI	BootULCI
*c* Social support → Procrastination (total effect)	-0.106	0.010	-0.126	-0.086
*c*’ Social support → Procrastination (direct effect)	-0.029	0.015	-0.058	0.001
*ab* Total indirect effect	-0.077	0.014	-0.104	-0.051
*a*_1_*b*_1_ Social support → Self-efficacy → Procrastination	-0.017	0.007	-0.032	-0.004
*a*_2_*b*_2_ Social support → Resilience → Procrastination	-0.047	0.012	-0.072	-0.025
*a*_1_*d*_21_b_2_ Social support → Self-efficacy → Resilience → Procrastination	-0.013	0.003	-0.020	-0.007

Note: SE = standard error, LLCI = lower limit confidence interval, ULCI = upper limit confidence interval

## Discussion

This study examined the multiple mediating roles of self-efficacy and resilience in the relationship between social support and procrastination among vocational college students. Our findings indicated that social support negatively affected procrastination through three indirect paths: the relationship mediated by self-efficacy, the relationship mediated by resilience, and the relationship sequentially mediated by self-efficacy and resilience. In other words, students with sufficient social support tended to exhibit better self-efficacy and resilience, which, in turn, was associated with lower procrastination tendency.

### The role of social support in procrastination

In the current study, we observed a negative association between social support and procrastination among vocational college students, consistent with previous research [11, 66]. One possible explanation is that social support received from intimate social networks can help enhance individuals’ problem-solving skills and social supervision, thereby facilitating the sustained execution of intended behaviour and reducing the likelihood of procrastination [12, 32]. In addition, social support may alter students’ perceptions of the challenges they encounter and give them greater confidence in achieving their goals and taking action, thereby reducing their tendency to procrastinate [11]. Therefore, it is crucial to focus on the impact of social support on procrastination, and our study offered a novel perspective for comprehending the impact of social support on procrastination through its indirect paths of self-efficacy and resilience.

### Mediating role of self-efficacy

The present study provided evidence for the mediating role of self-efficacy in the relationship between social support and procrastination among vocational college students. In other words, students with adequate social support demonstrated higher levels of self-efficacy, which in turn was linked to a lower level of procrastination. Our findings revealed the positive impact of social support on self-efficacy, consistent with prior research [38, 39]. This could be attributed to the role of social support in enhancing individuals’ sense of self-efficacy by providing resources, suggestions, knowledge for coping with adversity and challenging situations, as well as by encouraging and approving their abilities and behaviours [38], and nurturing their general sense of self-worth and personal control [39]. Furthermore, this study revealed a negative association between self-efficacy and procrastination, which replicated the findings of previous studies [9, 37], possibly because students with high self-efficacy tended to perceive challenging tasks as opportunities to be embraced, rather than as problems to be avoided, and were less likely to engage in procrastination when faced with obstacles [9]. These findings highlighted the important effect of self-efficacy on procrastination, particularly among students with inadequate social support. Interventions that focus on promoting self-efficacy may prevent or reduce procrastination among vocational college students.

### Mediating role of resilience

Our findings revealed that resilience acted as a mediator between social support and procrastination among vocational college students. In other words, students with higher social support levels reported better resilience, which in turn contributed to a lower tendency to procrastinate. Our results indicated that social support had a positive influence on self-efficacy, consistent with previous studies [67, 68]. When faced with adverse and stressful situations, students with more external support resources exhibit elevated levels of resilience [67]. Social support from family, friends, and specialists can buffer the negative influence of stressful experiences, enhance individuals’ social adaptability, and contribute to the development of elevated resilience [68]. Furthermore, the results demonstrated that more resilient students reported a lower tendency to procrastinate, in line with previous research [41, 45], possibly because resilient individuals typically exhibit more positive emotions and coping strategies to deal with stressful events [69] and tend to be capable of adapting quickly and finding solutions to their issues, which decreases their likelihood of procrastination [41]. Thus, resilience plays a crucial role in the relationship between social support and procrastination. Interventions focusing on enhancing resilience may help prevent or reduce procrastination among vocational college students, especially those with low levels of social support.

### Serial mediation role of self-efficacy and resilience

This study revealed that self-efficacy and resilience served as sequential mediators between social support and procrastination. In other words, higher levels of social support were sequentially associated with increased self-efficacy first and then enhanced resilience, which was, in turn, associated with less procrastination. Students with higher levels of social support are more likely to develop greater self-efficacy [70]. Moreover, we found a positive association between self-efficacy and resilience, consistent with previous research [48, 71]. This can be attributed to the significance of self-efficacy in enhancing individuals’ aspirations, analytical thinking, and perseverance when confronted with difficult circumstances, and subsequently fostering individuals’ capacity to adapt and navigate challenging situations with flexibility, thereby promoting their resilience [71]. Additionally, more resilient students display a greater tendency to approach challenging situations positively, which ultimately prevents or reduces procrastination [41]. Hence, the path from self-efficacy to resilience is an important bridge for the effect of social support on procrastination. Interventions designed to enhance self-efficacy and resilience may be beneficial for preventing or reducing procrastination among vocational college students, especially those with inadequate social support.

### Implications

On the basis of our findings, it is recommended that efforts to mitigate and prevent procrastination should be integrated into comprehensive educational strategies. Targeted interventions should involve the reinforcement of social support, as well as the enhancement of self-efficacy and resilience. First, educators and social organisations should establish a functional social support network, as well as cultivate students’ social skills and guide them in recognising and using various sources of social support [11]. These interventions intended to strengthen social support may indirectly prevent or reduce procrastination by fostering self-efficacy and resilience. Second, our findings highlight the importance of enhancing self-efficacy. Prior research has demonstrated the effectiveness of self-assessment interventions [72] and expectancy-related interventions [1], so promoting these approaches to enhancing self-efficacy is a future direction for preventing or reducing procrastination among vocational college students. Third, our findings underscore the significance of enhancing resilience, which may be achieved through the implementation of the Penn Resiliency Program, a group-based cognitive-behavioural intervention [73]. Furthermore, given the sequential mediating roles of self-efficacy and resilience in the relationship between social support and procrastination, interventions that target both factors will be more effective in preventing or reducing procrastination.

### Limitations

This study has some limitations. First, it was difficult to infer causality using the cross-sectional design, and longitudinal studies are needed to understand the causal relationships between the study variables. Second, convenience sampling from one vocational college limited generalisation. Future studies should collect data from multiple centres to increase the representativeness of the results. Third, we collected data using self-report measures; therefore, the results may be biased. Finally, we measured general procrastination; if particular kinds of procrastination are evaluated, the results may differ.

## Conclusions

Vocational college students who obtain less social support are more likely to engage in procrastination. Self-efficacy and resilience serve as multiple mediators between social support and procrastination, and interventions designed to enhance self-efficacy and resilience may directly prevent or reduce procrastination. In addition, procrastination may be indirectly prevented or reduced by enhancing social support among vocational college students.

## Data Availability

The data that support the findings of this study are available from the corresponding author upon reasonable request.
